# Outcomes of thrombolytic therapy in acute ischemic stroke: mothership, drip-and-ship, and ship-and-drip paradigms

**DOI:** 10.1186/s12883-020-1631-9

**Published:** 2020-02-03

**Authors:** Naruchorn Kijpaisalratana, Aurauma Chutinet, Wasan Akarathanawat, Pakkawan Vongvasinkul, Nijasri C. Suwanwela

**Affiliations:** 1 Division of Neurology, Department of Medicine, Faculty of Medicine, Chulalongkorn University, Rama IV Road, Bangkok, 10330 Thailand; 2 Chula Neuroscience Center, King Chulalongkorn Memorial Hospital, Thai Red Cross Society, Rama IV Road, Bangkok, 10330 Thailand; 3 Division of Academic Affairs, Faculty of Medicine, Chulalongkorn University, Rama IV Road, Bangkok, 10330 Thailand; 4 Chulalongkorn Stroke Center, King Chulalongkorn Memorial Hospital, Bangkok, Thailand

**Keywords:** Acute ischemic stroke, Thrombolytic, Telestroke, Mothership, Drip-and-ship, Ship-and-drip

## Abstract

**Background:**

Chulalongkorn Stroke Center is a comprehensive stroke center (CSC) located in Bangkok, Thailand. Our stroke network consists of different levels of spoke hospitals, ranging from community hospitals where thrombolytic treatment is not available, to those capable of onsite thrombolytic therapy. This study aimed to assess the time to treatment and outcomes among acute ischemic stroke patients who received thrombolytic treatment in the Chulalongkorn Stroke Network by 1.) Direct arrival at the CSC (mothership) 2.) Telestroke-assisted thrombolytic treatment with secondary transfer to the CSC (drip-and-ship) 3.) Referral from community hospital to the CSC for thrombolytic treatment (ship-and-drip).

**Methods:**

Acute ischemic stroke patients who received thrombolytic treatment during January 2016–December 2017 in the Chulalongkorn Stroke Network were studied. Time to treatment and clinical outcomes were compared among treatment groups.

**Results:**

There were 273 patients in the study including 147, 87, and 39 patients in mothership, drip-and-ship, and ship-and-drip paradigms, respectively. The door-to-needle-time (DTN) and onset-to-needle-time (OTN) times were significantly longest in ship-and-drip group (146.5 ± 62/205.03 ± 44.88 mins) compared to mothership (38 ± 23/155.2 ± 60.54 mins) and drip-and-ship (63.0 ± 44/166.09 ± 87 mins), *P* < 0.05. There was no significant difference regarding functional independence defined by modified Rankin Scale (mRS) ≤ 2 at 3 months (*P* = 0.12), in-hospital mortality (*P* = 0.37), mortality at 3 months (*P* = 0.73), and symptomatic intracerebral hemorrhage (*P* = 0.24) among groups.

**Conclusion:**

Thrombolytic treatment with drip and ship method under teleconsultation is feasible in Thailand. There was no difference of clinical outcome among the 3 treatment paradigms. However, DTN time and OTN time were longest in the ship-and-drip paradigm.

## Background

Stroke is the leading cause of death and disability in Thailand and worldwide [1]. Intravenous thrombolytic therapy with recombinant tissue plasminogen activator (rtPA) is the standard treatment of acute ischemic stroke [2]. Despite the proven efficacy of thrombolytic therapy, the rate of patients receiving intravenous thrombolysis is relatively low especially in neurologically underserved areas [3].

In Thailand, the first thrombolytic treatment for acute ischemic stroke was given at King Chulalongkorn Memorial Hospital in 1996 [4]. The Ministry of Public Health and the National Health Security Office (NHSO) had accepted the use of intravenous thrombolysis as the standard treatment of acute ischemic stroke in 2008. Therefore, the cost of the treatment can be totally reimbursed for all Thai citizens by 3 main payers including the universal coverage program by NHSO, the Social Security Office, and the Comptroller General’s Department for the government officers [5]. Since then the stroke fast track system has been implemented throughout the country. The national thrombolytic treatment for acute ischemic stroke rate has been increased from 0.38% in 2008 [1] to 4.36% in 2015 [6]. Despite the increasing number of patients receiving thrombolytic treatment, this figure is relatively low compared to the number of all ischemic stroke patients. The complexity of the stroke fast track system, which requires multidisciplinary team, management in a timely manner, and lack of stroke specialists limits the availability of thrombolytic treatment in Thailand mainly to provincial and university hospitals.

The initiation of a telestroke service under the guidance of stroke specialists in a comprehensive stroke center has increased the use of thrombolytic therapy among community hospitals where stroke specialists are lacking [7]. According to the American Heart Association/American Stroke Association (AHA/ASA), telestroke facilitates the use of thrombolytic therapy in acute ischemic stroke with similar safety as the primary stroke centers [8].

Chulalongkorn Stroke Network consists of different levels of spoke hospitals, ranging from community hospitals where thrombolytic treatment is not available, to those capable of onsite thrombolytic therapy. The aim of this study is to evaluate time to treatment and clinical outcomes of patients receiving thrombolytic treatment among different thrombolytic delivery protocol in the Chulalongkorn Stroke Network.

## Methods

### Study design

This is a retrospective observational study approved by the Institutional Review Board of the Faculty of Medicine, Chulalongkorn University. Medical records of all patients with acute ischemic stroke receiving thrombolytic therapy during January 2016–December 2017 were reviewed. Chulalongkorn Stroke Network has 25 spoke hospitals with distance to hub hospital ranging from 1 to 60 km (average 19 km), and referral time ranging from 5 to 60 min (average 23 min). Patients were divided into 3 groups including 1.) Patients directly arriving at Chulalongkorn Comprehensive Stroke Center (CSC) (“mothership” protocol) 2.) Patients receiving intravenous thrombolytic therapy via telestroke consultation at the spoke hospital with secondary transfer to the CSC (“drip-and-ship” protocol) 3.) Patients who presented at the community hospital with secondary transfer to the CSC for thrombolytic treatment (“ship-and-drip” protocol). Informed consent for intravenous rtPA was obtained in all cases. Computed tomography (CT) was performed at 24 h after thrombolytic treatment or earlier in case of neurological worsening. All patients receiving thrombolytic treatment in the Chulalongkorn Stroke Network had 3 months follow up at the stroke clinic at King Chulalongkorn Memorial Hospital. Data consisting of National Institute of Health Stroke Scale (NIHSS), functional outcome assessed by modified Rankin Scale (mRS), and mortality were reviewed in the medical records from the stroke clinic.

### Inclusion&exclusion criteria

All patients with acute ischemic stroke receiving thrombolytic treatment during January 2016–December 2017 in the Chulalongkorn Stroke Network were included in the study. No patient was excluded in the analysis. In drip-and-ship protocol, patients with acute ischemic stroke ≥18 years of age without hemorrhagic stroke in CT scan who presented at the spoke hospital within 4.5 h after onset and eligible for intravenous thrombolytic therapy were included in the study. Consent for telestroke consultation was obtained in every patient.

### Thrombolytic treatment

#### Mothership

Patients with acute ischemic stroke who presented at the emergency room of the King Chulalongkorn Memorial Hospital within 4.5 h after onset were triaged by the nursing staff. Emergency physicians then initially assessed patients and activated the “Stroke Fast Track” program once the diagnosis of stroke was suspected. The on-call neurologist was immediately notified. Non-contrast computed tomography (CT) of the brain and essential blood tests were promptly performed. The decision of thrombolysis was made by the on-call neurologist according to the inclusion and exclusion criteria from the AHA/ASA [8] in agreement with the decision of patients and their family.

#### Drip-and-ship and Telestroke system

When patients with acute ischemic stroke arrived at the spoke hospital where thrombolytic treatment was available within 4.5 h after onset, emergency physicians of the spoke hospital assessed patients and immediately notified the stroke specialist at the Chulalongkorn CSC via an electronic message or telephone call. Telestroke consultation was then initiated with real-time interactive videoconferencing. The stroke specialist was able to perform the physical examination assisted by the onsite physician at the spoke hospital. Neuroimaging was reviewed via teleradiology. The decision for thrombolysis was made by the onsite physician undersupervision of the stroke specialist after telestroke consultation. The onsite physician discussed the risk and benefit of the thrombolytic treatment with the patients and their family. Once the decision for thrombolysis was made, rtPA was given at the spoke hospital. Intravenous rtPA standard dose (0.9 mg/kg over 60 min with initial 10% of dose bolus over 1 min) was given to the patient [2]. Patients were then transferred to the Chulalongkorn CSC for further neurovascular investigation and potential of endovascular intervention. The rtPA infusion was continued during transportation to the stroke center. Registered nurse working in the ambulance service accompanied the patient en route to the stroke center to closely monitor the patient. During transportation, the nurse could access the telestroke consultation with the stroke center. Patient’s history, physical examination, along with initial assessment of NIHSS were evaluated by onsite physician. The case file was transferred along with the patient to the CSC.

#### Ship-and-drip

When a stroke patient presented at the spoke hospital where thrombolytic therapy was not available, the on-call neurologist at the Chulalongkorn CSC was immediately notified. Non-contrast CT scan of the brain was performed at the spoke hospital. After initial assessment by the onsite physician at the spoke hospital, the patient was immediately transferred to the Chulalongkorn CSC for thrombolytic treatment. The on-call neurologist notified the stroke fast track team consisting of emergency medicine physician and nursing staff at the emergency department as well as the neuroradiologist and neuroimaging technician to standby for referral patients. CT scan was repeated if the timing of the initial CT scan was longer than 1 h upon arrival at the Chulalongkorn CSC according to the referral protocol by the Chulalongkorn CSC. Repeated CT scan was performed in order to ensure that there was no contraindication based on neuroimaging study such as clear hypoattenuation especially in those referred from the long-distance spoke hospitals. The on-call neurologist then made the decision of thrombolysis after discussion with the patients and their family.

### Outcome measures

Baseline characteristics including gender, age, initial NIHSS, and underlying diseases were collected. The clinical outcome of functional independence defined by modified Rankin Scale (mRS) ≤ 2 at 3 month were evaluated. Safety outcomes were in-hospital mortality, mortality at 3 months, and symptomatic intracerebral hemorrhage (sICH) defined by the Safe Implementation of Thrombolysis in Stroke-Monitoring Study (SITS-MOST) criteria [9].

### Statistical analysis

SPSS 22.0 for Mac software package (SPSS, Inc., Chicago, IL) was used for all analyses. Kolmogorov-Smirnov test was used to determine the distribution of the data. Baseline characteristics were expressed in mean +/− standard deviation (SD) or median with interquartile range (IQR). Comparisons of continuous variables between the 3 groups were performed by one-way analysis of variance or Kruskal Wallis test. Categorical variables were presented in percentages and frequencies. Pearson’s Chi square or Fisher’s exact test was used for categorical variables comparison among the 3 groups. Binary logistic regression was performed to identify factors that associated with favorable clinical and safety outcomes. Factors with *P* value < 0.1 in the univariable analysis were included in the multivariable logistic regression. A P value of 0.05 was considered statistically significant.

## Results

### Baseline characteristics

During January 2016–December 2017, there were 1608 acute ischemic stroke patients admitted at the King Chulalongkorn Memorial Hospital, 273 (17%) patients received intravenous rtPA. Of these, 147 patients presented directly at Chulalongkorn CSC and received thrombolytic treatment under “mothership” protocol, 87 patients received thrombolytic treatment under “drip-and-ship” protocol via telestroke consultation, and 39 patients received intravenous rtPA at the Chulalongkorn CSC under “ship-and-drip” protocol. There was no significant difference among the 3 treatment groups regarding age, gender, initial NIHSS, risk factors, and stroke subtypes by TOAST classification [10] (Table 1). There were 24 patients (8.8%) who underwent mechanical thrombectomy after receiving thrombolytic treatment.

**Table 1:** Baseline characteristics of patients receiving thrombolytic treatment in mothership, drip-and-ship, and ship-and-drip paradigms

	Overall (*n* = 273)	Mothership (*n* = 147)	Drip-and-Ship (*n* = 87)	Ship-and-Drip (*n* = 39)	*P* value
Age, med (IQR)	66 (22)	68 (22)	64 (22)	60 (29)	0.22
Female, n (%)	123 (45.1%)	71 (48.3%)	34 (39.1%)	18 (46.2%)	0.39
Initial NIHSS, med (IQR)	10 (11)	8 (10)	11 (13)	12 (12)	0.18
Risk factors, n (%)					
- Hypertension	160 (58.6%)	84 (57.1%)	50 (57.5%)	26 (66.7%)	0.54
- Diabetes mellitus	71 (26.0%)	36 (24.5%)	23 (26.4%)	12 (30.8%)	0.73
- Dyslipidemia	99 (36.3%)	58 (39.5%)	24 (27.6%)	17 (43.6%)	0.11
- History of stroke	42 (15.4%)	28 (19.0%)	11 (12.6%)	3 (7.7%)	0.15
- History of myocardial infarction	25 (9.2%)	17 (11.6%)	6 (6.9%)	2 (5.1%)	0.31
Underlying malignancy	19 (7.0%)	15 (10.2%)	2 (2.3%)	2 (5.1%)	0.06
Stroke subtypes, n (%)					0.07
- Large vessel	38 (13.9%)	17 (11.6%)	20 (23.0%)	1 (2.6%)	
- Small vessel	55 (20.1%)	34 (23.1%)	15 (17.2%)	6 (15.4%)	
- Cardioembolic	99 (36.3%)	52 (35.4%)	29 (33.3%)	18 (46.2%)	
- Other determined	2 (0.7%)	2 (1.4%)	0 (0%)	0 (0%)	
- Undetermined	79 (28.9%)	42 (28.6%)	23 (26.4%)	14 (35.9%)	
Thrombectomy	24 (8.8%)	13 (8.8%)	10 (11.5%)	1 (2.6%)	0.26

### Time to treatment

**Fig. 1:**
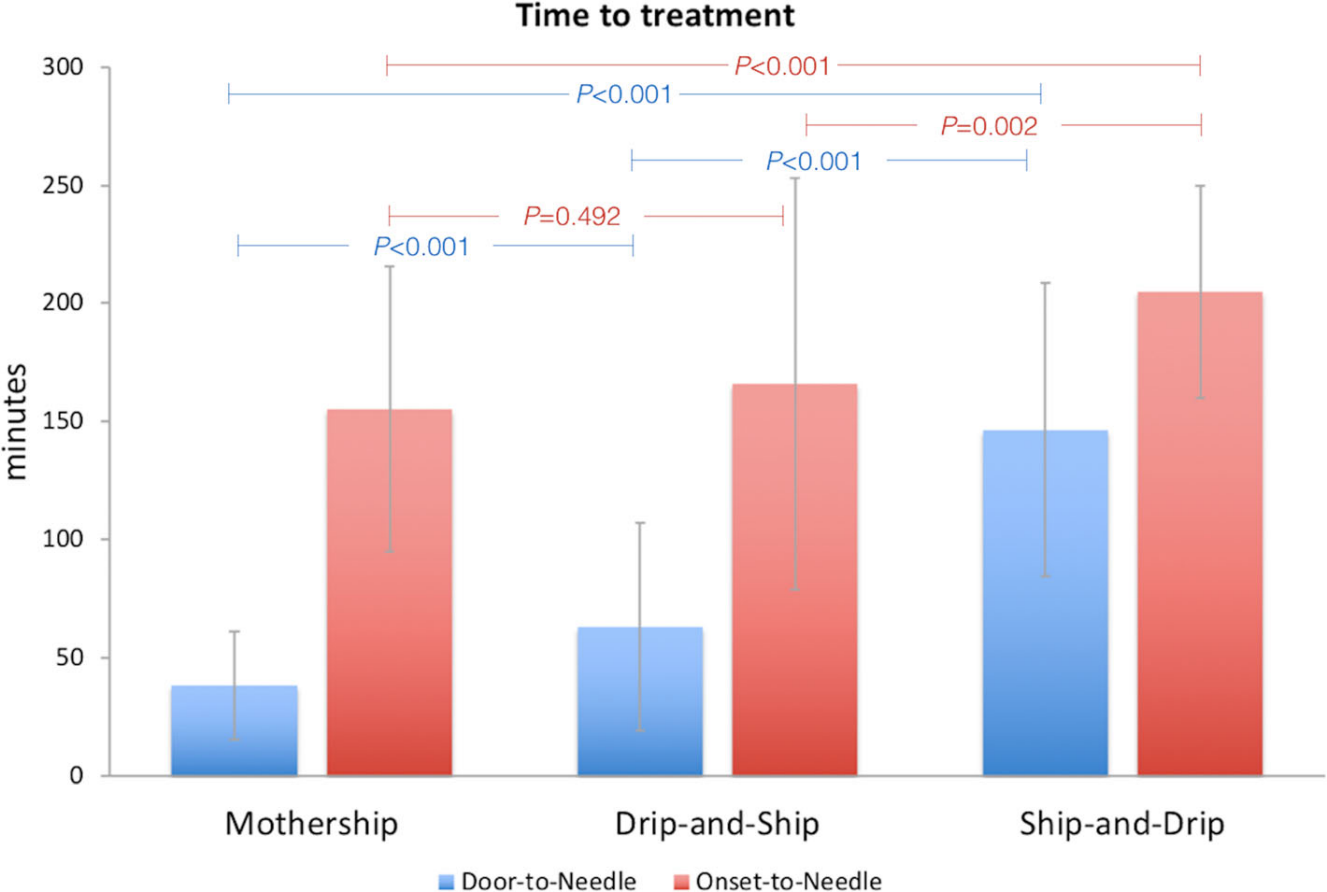
Time to treatment according to mode of rtPA delivery

DTN times were significantly longest in ship-and-drip group (146.5 ± 62 mins) compared to mothership (38 ± 23 mins), *P* < 0.001 and drip-and-ship (63 ± 44 mins), *P* < 0.001. In addition, OTN times were also significantly longest in ship-and-drip group (205 ± 44.88 mins) compared to mothership (155.2 ± 60.54 mins); mean difference ± SEM = 49.8 ± 10.38; *P* < 0.001 and drip-and-ship (166.09 ± 87 mins); mean difference ± SEM = 38.93 ± 11.10; *P* = 0.002 (Fig. 1).

### Clinical and safety outcomes

**Fig. 2:**
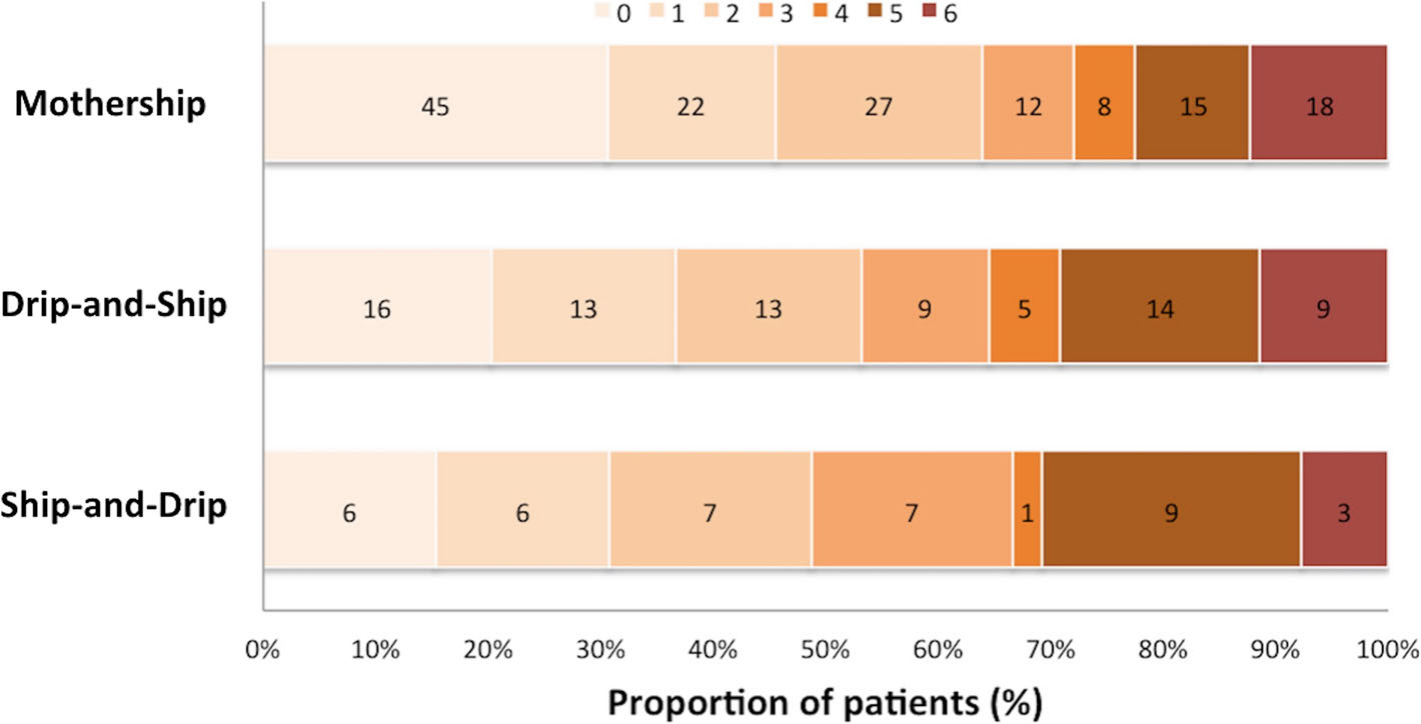
Scores on the modified rankin scale at 90 days according to mode of rtPA delivery

Table 2 demonstrates clinical and safety outcomes of patients receiving thrombolytic treatment. There was no statistical significance in functional independence at 3 months among patients receiving thrombolytic treatment in mothership (63.9%) and those in drip-and-ship (53.2%), and ship-and-drip (48.7%) (*P* = 0.12) **(**Fig. 2 and Table 2). Among patients undergoing mechanical thrombectomy, there were 9 patients (37.5%) achieving functional independence at 3 months including 7 patients from mothership and 2 patients from drip-and-ship group.

**Table 2:** Clinical and safety outcomes in patients receiving thrombolytic treatment in mothership, drip-and-ship, and ship-and-drip paradigms

	Overall	Mothership	Drip-and-Ship	Ship-and-Drip	*P* Value
Functional Independence, n(%)	155 (58.5%)	94 (63.9%)	42 (53.2%)	19 (48.7%)	0.12
In-hospital Mortality, n(%)	17 (6.4%)	12 (8.2%)	4 (5.0%)	1 (2.6%)	0.37
Mortality at 3 months, n(%)	30 (11.3%)	18 (12.2%)	9 (11.4%)	3 (7.7%)	0.73
sICH, n(%)	14 (5.3%)	5 (3.4%)	6 (7.6%)	3 (7.7%)	0.24

The overall in-hospital mortality rate and mortality rate at 3 months was 6.4 and 11.3% respectively. There was no significant difference regarding in-hospital mortality (*P* = 0.37) and mortality at 3 months (*P* = 0.73) among all groups. The in-hospital mortality rate among patients undergoing mechanical thrombectomy was 8.3%. Two of them were in the mothership group.

The overall rate of sICH was 5.3%. There was no significant difference regarding the rate of sICH among treatment groups. Cryoprecipitate and tranexamic acid were given to patients who had sICH within 24 h after intravenous rtPA administration along with neurosurgical consultation. Among 14 patients with sICH, 8 patients underwent decompressive craniectomy. The in-hospital mortality rate among patients with sICH was 28.6%.

In multivariable logistic regression, age (OR 0.98; 95% CI 0.954–0.998; *P* = 0.032), initial NIHSS (OR 0.82; 95% CI 0.769–0.870; *P* = < 0.001), OTN time (OR 0.99; 95% CI 0.988–0.999; *P* = 0.019), and underlying of malignancy (OR 0.18; 95% CI 0.044–0.733; *P* = 0.017) were factors associated with functional independence at 3 months. Female sex (OR 8.41; 95% CI 1.788–39.532; *P* = < 0.007) and initial NIHSS (OR 1.20; 95% CI 1.091–1.308; *P* = < 0.001) were factors associated with in-hospital mortality. Factors associated with mortality at 3 months were female sex (OR 3.73; 95% CI 1.447–9.611; *P* = 0.006), initial NIHSS (OR 1.15; 95%CI 1.076–1.229; *P* < 0.001), OTN time (OR 1.01; 95% CI 1.001–1.016; *P* = 0.034), and underlying of malignancy (OR 8.24; 95% CI 2.491–27.271; *P* = 0.001). Only OTN time (OR 1.02; 95% CI 1.005–1.029; *P* = 0.006) and history of myocardial infarction (OR 7.21; 95% CI 1.861–27.927; *P* = 0.004) were factors associated with sICH. The mode of rtPA delivery including mothership, drip-and-ship, and ship-and-drip was not a significant predictor regarding post-thrombolytic favorable clinical and safety outcomes.

## Discussion

Intravenous rtPA treatment has been shown to improve clinical outcome among patients with acute ischemic stroke [11, 12]. Telestroke facilitates acute stroke care by connecting onsite physicians with stroke experts to help increase use of thrombolytic treatment [13] and improve the number of acute ischemic stroke patients receiving effective treatment [14]. Drip-and-ship rtPA treatment paradigm has been shown to contribute to an increase in the rate of thrombolytic treatment in a nationwide study [15, 16].

Due to the diversity of the spoke hospitals, our stroke network has different paradigms for thrombolytic delivery suitable for each spoke hospital. Our study is the first to demonstrate the time to treatment and clinical outcomes among patients treated with three different mode of rtPA delivery including mothership, drip-and-ship, and ship-and-drip paradigms. Most studies have compared mothership with drip-and-ship [15–21] or mothership with ship-and-drip [22–26].

In our study, there were significant differences in OTN and DTN times among treatment protocols. Patients in ship-and-drip protocol had the longest OTN and DTN times which was consistent with previous studies [24–26].

Our study demonstrated a non-significant trend towards the functional independence at 3 months defined by mRS ≤ 2 among mothership and drip-and-ship groups. A single center retrospective study showed similar functional outcome of excellent mRS defined by mRS ≤ 1 between patients in mothership and those in the drip-and-ship protocol [19]. In a retrospective nationwide study using Get With the Guidelines (GWTG) Stroke database, lower mRS at the follow up period were similar among patients receiving thrombolytic treatment at the regional stroke center and patients in drip-and-ship group [20].

When comparing between mothership and ship-and-drip protocol, clinical outcomes were conflicting among studies. This might be due to the different definition for good clinical outcome. Mothership protocol was associated with good outcome defined by mRS ≤ 2 at 3 months after adjustment for stroke severity and baseline variables [23, 25]. However, a study by Merino et al. showed that clinical improvement defined by an improvement of NIHSS > 4 points at 3 months was similar among patients treated with thrombolytic in mothership and ship-and-drip protocol [24]. In addition, clinical improvement defined by NIHSS reduction ≥4 at discharge combined with mRS ≤ 1 at 3 months was similar in both treatment groups [26].

Regarding safety outcomes in our study, in-hospital mortality, mortality at 3 months, and symptomatic ICH were similar among all treatment groups. The overall 3- month mortality rate of 12.4% in our study was comparable to those reported in the clinical trials [9, 11]. In-hospital mortality rate was found to be similar between patients in mothership and drip-and-ship protocol [17–19]. However, results from the nationwide study showed slightly higher in-hospital mortality among drip-and-ship patients compared to patients in mothership protocol (10.93 vs. 9.67, *P* = 0.0002) [16]. Mortality rate at 3 months was found to be similar between mothership and ship-and-drip protocol [24]. In addition to the initial NIHSS, female sex was a factor associated with both in-hospital mortality and mortality at 3 months in multiple logistic regression. However, studies regarding gender and mortality outcome in thrombolytic treatment reported conflicting results. One stroke registry reported that male gender was associated with in-hospital death [27], whereas sex was not associated with in-hospital [27–29] and 3-month mortality [30, 31] in other studies.

The rate of symptomatic intracerebral hemorrhage was similar when comparing mothership and drip-and-ship protocol [18–20] and mothership and ship-and-drip protocol [23, 26]. However, in the GWTG stroke registry, sICH was found to be slightly higher among patients with drip-and-ship protocol compared to front-door patients (5.79% vs. 5.22%, *P* = 0.001) [16]. In addition, a retrospective study from the ischemic stroke registry in Spain revealed that sICH was found to be more frequent in ship-and-drip patients compared to mothership patients (14% vs 4.7%, *P* = 0.04) [25]. In multiple logistic regression, our study demonstrated that history of myocardial infarction was another risk factor for sICH in addition to OTN time. Antiplatelet use either single or dual in patients with myocardial infarction might contributed to sICH in our study population.

In order to improve the stroke network, neurovascular investigation by CT angiography and CT perfusion to identify patients who are candidates for mechanical thrombectomy should be developed at thrombolysis-capable spoke hospitals. Therefore, patients without large vessel occlusion can stay at the spoke hospital for post thrombolytic care without transferring to the CSC. From our experience, mechanical thrombectomy was performed in 10 out of 87 patients (11.5%) in drip-and-ship paradigm. Spoke hospitals that are unable to give thrombolysis should be encouraged to set up a stroke fast track program by engaging a multidisciplinary team to facilitate thrombolytic delivery assisted by telestroke under the supervision of the CSC.

Due to a relatively small number of patients in each study group the results should be interpreted with caution. Our study did not demonstrate the outcome difference regarding functional independence among treatment paradigms despite the significant difference in time to treatment both DTN and OTN. A relatively small number of patients in each group might result in non-significant difference in both clinical and safety outcomes. In addition, the study design is an observational study, which may not be powered to detect significant clinical and safety outcome differences. Selection bias among ship-and-drip group should be taken into account. Hospitals in this group are usually small hospitals with fewer facilities. Lack of experienced healthcare providers may impact patient management and clinical outcome. Patients with severe ischemic stroke who arrived at the hospital far from the comprehensive center where referrals cannot be done within thrombolytic window may not have been included in the study.

## Conclusion

Telestroke assisted thrombolytic treatment prior to referral to the stroke center, and thrombolytic treatment at the stroke center in patients transferred from the community hospitals, were as effective as treatment given at the CSC with the similar safety profile. However, the DTN time and OTN time were significantly shortest in the mothership group.

## Data Availability

The datasets are available from the corresponding author on reasonable request.

## Abbreviations

AHA/ASA: American Heart Association/American Stroke Association
CSC: comprehensive stroke center
CT: computed tomography
DTN: door-to-needle time
GWTG: Get With The Guidelines
IQR: interquartile range
mRS: modified Rankin Scale
NIHSS: National Institute of Health Stroke Scale
OTN: onset-to-needle time
rtPA: recombinant tissue plasminogen activator
SD: standard deviation
sICH: symptomatic intracerebral hemorrhage
SITS-MOST: Safe Implementation of Thromgolysis in Stroke-Monitoring Study
TOAST: Trial of Org 10,172 in Acute Stroke Treatment

## References

[1] Suwanwela NC (2014). Stroke epidemiology in Thailand. J Stroke.

[2] Powers William J, Rabinstein Alejandro A, Ackerson T, Adeoye Opeolu M, Bambakidis Nicholas C, Becker K (2018). 2018 guidelines for the early management of patients with acute ischemic stroke: a guideline for healthcare professionals from the American Heart Association/American Stroke Association. Stroke.

[3] Kepplinger J, Barlinn K, Deckert S, Scheibe M, Bodechtel U, Schmitt J (2016). Safety and efficacy of thrombolysis in telestroke: a systematic review and meta-analysis. Neurology.

[4] Suwanwela NC, Phanthumchinda K, Suwanwela N, Tantivatana J, Janchai A (2001). Thrombolytic treatment for acute ischemic stroke: a 2 year-experience at King Chulalongkorn Memorial Hospital. J Med Assoc Thail.

[5] Suwanwela NC, Chutinet A, Kijpaisalratana N (2018). Thrombolytic treatment in Thailand. J Stroke Med.

[6] Tiamkao S (2015). Stroke fast track in Thailand 2558. Thai Neuro J.

[7] Muller-Barna P, Schwamm LH, Haberl RL (2012). Telestroke increases use of acute stroke therapy. Curr Opin Neurol.

[8] Jauch EC, Saver JL, Adams HP, Bruno A, Connors JJ, Demaerschalk BM (2013). Guidelines for the early management of patients with acute ischemic stroke: a guideline for healthcare professionals from the American Heart Association/American Stroke Association. Stroke.

[9] Wahlgren N, Ahmed N, Dávalos A, Ford GA, Grond M, Hacke W (2007). Thrombolysis with alteplase for acute ischaemic stroke in the safe implementation of thrombolysis in stroke-monitoring study (SITS-MOST): an observational study. Lancet.

[10] Adams HP, Bendixen BH, Kappelle LJ, Biller J, Love BB, Gordon DL (1993). Classification of subtype of acute ischemic stroke. Definitions for use in a multicenter clinical trial. TOAST. Trial of org 10172 in acute stroke treatment. Stroke.

[11] Disorders TNIoN, Group Sr-PSS (1995). Tissue plasminogen activator for acute ischemic stroke. N Engl J Med.

[12] Chiu D, Krieger D, Villar-Cordova C, Kasner SE, Morgenstern LB, Bratina PL (1998). Intravenous tissue plasminogen activator for acute ischemic stroke: feasibility, safety, and efficacy in the first year of clinical practice. Stroke.

[13] Meyer BC, Demaerschalk BM (2012). Telestroke network fundamentals. J Stroke Cerebrovasc Dis.

[14] Levine SR, Gorman M (1999). “Telestroke” : the application of telemedicine for stroke. Stroke.

[15] Tekle WG, Chaudhry SA, Hassan AE, Rodriguez GJ, Suri MFK, Qureshi AI (2012). Drip-and-ship thrombolytic treatment paradigm among acute ischemic stroke patients in the United States. Stroke.

[16] Sheth KN, Smith EE, Grau-Sepulveda MV, Kleindorfer D, Fonarow GC, Schwamm LH (2015). Drip and ship thrombolytic therapy for acute ischemic stroke: use, temporal trends, and outcomes. Stroke.

[17] Ionita CC, Sharma J, Janicke DM, Levy EI, Siddiqui AH, Agrawal S (2009). Acute ischemic stroke and thrombolysis location: comparing telemedicine and stroke center treatment outcomes. Hosp Pract.

[18] Mansoor S, Zand R, Al-Wafai A, Wahba MN, Giraldo EA (2013). Safety of a “drip and ship” intravenous thrombolysis protocol for patients with acute ischemic stroke. J Stroke Cerebrovasc Dis.

[19] Martin-Schild S, Morales MM, Khaja AM, Barreto AD, Hallevi H, Abraham A (2011). Is the drip-and-ship approach to delivering thrombolysis for acute ischemic stroke safe?. J Emerg Med.

[20] Pervez MA, Silva G, Masrur S, Betensky RA, Furie KL, Hidalgo R (2010). Remote supervision of IV-tPA for acute ischemic stroke by telemedicine or telephone before transfer to a regional stroke center is feasible and safe. Stroke.

[21] Wang DZ, Rose JA, Honings DS, Garwacki DJ, Milbrandt JC (2000). Treating acute stroke patients with intravenous tPA. The OSF stroke network experience. Stroke.

[22] de la Ossa NP, Sanchez-Ojanguren J, Palomeras E, Millan M, Arenillas JF, Dorado L (2008). Influence of the stroke code activation source on the outcome of acute ischemic stroke patients. Neurology.

[23] Kim DH, Cha JK, Park HS, Choi JH, Kang MJ, Huh JT (2014). Direct access to a hospital offering intravenous thrombolysis therapy improves functional outcome of acute ischemic stroke patients. J Clin Neurosci.

[24] Merino JG, Silver B, Wong E, Foell B, Demaerschalk B, Tamayo A (2002). Extending tissue plasminogen activator use to community and rural stroke patients. Stroke.

[25] Perez de la Ossa N, Millan M, Arenillas JF, Sanchez-Ojanguren J, Palomeras E, Dorado L (2009). Influence of direct admission to comprehensive stroke centers on the outcome of acute stroke patients treated with intravenous thrombolysis. J Neurol.

[26] Ribo M, Molina CA, Pedragosa A, Sanclemente C, Santamarina E, Rubiera M (2008). Geographic differences in acute stroke care in Catalunya: impact of a regional interhospital network. Cerebrovasc Dis.

[27] Tong X, George MG, Yang Q, Gillespie C (2014). Predictors of in-hospital death and symptomatic intracranial hemorrhage in patients with acute ischemic stroke treated with thrombolytic therapy: Paul Coverdell acute stroke registry 2008-2012. Int J Stroke.

[28] Heuschmann PU, Kolominsky-Rabas PL, Roether J, Misselwitz B, Lowitzsch K, Heidrich J (2004). Predictors of in-hospital mortality in patients with acute ischemic stroke treated with thrombolytic therapy. JAMA.

[29] Bateman Brian T, Schumacher HC, Boden-Albala B, Berman Mitchell F, Mohr JP, Sacco Ralph L (2006). Factors associated with in-hospital mortality after Administration of Thrombolysis in acute ischemic stroke patients. Stroke.

[30] Çetiner M, Aydin HE, Güler M, Canbaz Kabay S, Zorlu Y (2018). Predictive factors for functional outcomes after intravenous thrombolytic therapy in acute ischemic stroke. Clin Appl Thromb Hemost.

[31] Forster A, Gass A, Kern R, Wolf ME, Ottomeyer C, Zohsel K (2009). Gender differences in acute ischemic stroke: etiology, stroke patterns and response to thrombolysis. Stroke.

